# Supplementary material for: The genotype of barley cultivars influences multiple aspects of their associated microbiota via differential root exudate secretion
Source: PLoS Biol. 2024 Apr 25;22(4):e3002232. doi: 10.1371/journal.pbio.3002232 (PMC11045101; doi:10.1371/journal.pbio.3002232)
Supplement: S2 Table — (DOCX) [file pbio.3002232.s002.docx]

**S2 Table.** GC-MS detected compounds in root exudates of Tipple and Chevallier.

| **ID in heatmap** | **Compound** | **log2 normalised abundance Chevallier** | **log2 normalised abundance Tipple** | **Retention time (min)** |
| --- | --- | --- | --- | --- |
| 1 | L-sorbose 1 | 2.49938223 | -0.231661633 | 17.069666 |
| 2 | GABA | -9.815265743 | 0.641025063 | 13.264 |
| 3 | Malonic acid | 3.5084027 | 1.817025833 | 7.781 |
| 4 | Glycolic acid | -1.504392333 | 1.8737562 | 7.083 |
| 5 | Citric acid | 4.568336967 | -0.797406667 | 16.513334 |
| 6 | Phosphoric acid 1 | -1.3398835 | 2.073309533 | 20.1535 |
| 7 | Threonic acid | 4.716764651 | 3.53921329 | 13.575 |
| 8 | L-allothreonine | 4.716764651 | 3.623256957 | 10.199 |
| 9 | 1,6-anhydro-glucose | 4.716764651 | 3.44443829 | 18.16 |
| 10 | L-(+) Lactic acid | 3.622786647 | -0.015575407 | 6.8491664 |
| 11 | L-glutamic acid 1 | 3.029254833 | -2.867372333 | 13.1585 |
| 12 | 3-Phenyllactic acid | -4.7703856 | -0.635266653 | 26.257 |
| 13 | D-allose | -4.712648667 | 0.684558547 | 17.2145 |
| 14 | Palmitic acid | 5.170635893 | -1.299845383 | 18.850832 |
| 15 | Beta-alanine | 4.722871007 | -1.012701973 | 10.401333 |
| 16 | Ribitol | -2.40509 | 2.677856233 | 15.431 |
| 17 | Arabitol | 4.716764651 | 3.366139623 | 24.252 |
| 18 | L-alanine | -9.159545667 | 0.333281197 | 7.4663334 |
| 19 | Conduritol epoxide | 0.353543267 | -5.0805177 | 19.23975 |
| 20 | Gluconic acid | 4.716764651 | 3.475129123 | 18.357 |
| 21 | Oxalic acid | 5.272427253 | -1.202294853 | 8.008 |
| 22 | D-threitol | -2.40509 | 2.8156452 | 15.301001 |
| 23 | D-glucose | -2.40509 | 4.388778667 | 17.418999 |
| 24 | Myo-Inositol | 4.716764651 | 3.152618623 | 22.062 |
| 25 | Eicosapentaenoic acid | -1.437068157 | 0.183289687 | 23.783398 |
| 26 | D-mannose | 0.776330933 | -5.62717 | 17.35175 |
| 27 | L- sorbose 2 | -5.235006367 | -3.934811833 | 17.149668 |
| 28 | L-serine | 0.53474649 | -5.23333638 | 9.688499 |
| 29 | Petunidin-3-glucoside | -4.394409887 | 0.234955477 | 24.719002 |
| 30 | 3,4-MDEA | -2.187237467 | 2.1738755 | 8.4939995 |
| 31 | Acetohydroxamic acid | 5.029121063 | -0.729463073 | 7.6575 |
| 32 | Pelargonic acid | 5.324652977 | -0.80386369 | 11.103667 |
| 33 | Glycine | 0.5519614 | -5.265118043 | 10.355749 |
| 34 | Succinic acid | 4.157674699 | -0.149181683 | 10.451667 |
| 35 | Ethanolamine | 5.145020377 | -1.02423032 | 9.310667 |
| 36 | Tris(trimethylsilyl) phosphate | -9.3816611 | 0.330671787 | 9.90975 |
| 37 | Fumaric acid | -1.913231033 | 1.80298 | 10.9435005 |
| 38 | 4-Methylpentyl ethylphosphonofluoridate | 4.716764651 | 3.19421229 | 21.217 |
| 39 | Carboxylic Acid | 4.572312937 | -0.747330973 | 8.389 |
| 40 | Norvaline | 0.901025333 | -5.236252333 | 8.4945 |
| 41 | Propanamine | -2.40509 | 3.245493667 | 13.335501 |
| 42 | Methylmalonic acid | 4.716764651 | 3.464588957 | 8.063 |
| 43 | D-malic acid | 3.598440187 | -0.300637723 | 12.716499 |
| 44 | 6-hydroxy caproic acid | 4.716764651 | 3.500548957 | 14.888 |
| 45 | Glyceric acid | 0.618460973 | -0.663971903 | 10.646601 |
| 46 | D-sphingosine | 4.74252344 | -1.04045834 | 19.401833 |
| 47 | Digalacturonic acid | -4.684176667 | 0.165820123 | 21.686 |
| 48 | Caprylic acid | 4.716764651 | 3.383839257 | 16.31 |
| 49 | D-erythrose-4-phosphate | 4.716764651 | 3.673937923 | 15.898 |
| 50 | Acetylisatin | 2.393156 | -2.738779167 | 11.715 |
| 51 | Pipecolic acid | 4.716764651 | 3.599649257 | 13.158 |
| 52 | D (+) galactose | 2.970655467 | -3.0802461 | 17.384499 |
| 53 | Sedoheptulose | 9.738697433 | -1.041544583 | 18.161 |
| 54 | Tert-butyl isocyanate | 9.5147271 | -1.041544583 | 13.295 |
| 55 | Phosphoric acid 2 | 16.47379167 | -12.613472 | 9.893 |
| 56 | L-glutamic acid 2 | 7.876965933 | -8.069982433 | 13.293 |
| 57 | Sarcosine | 4.716764651 | 3.3304161 | 9.693 |
| 58 | Allo-inositol | 4.716764651 | 3.339910767 | 19.237 |
| 59 | m-toluic acid | 10.14417468 | -0.94812838 | 26.257 |
| 60 | 73.1@9.9 | 4.716764651 | 6.369109257 | 9.9 |
| 61 | 212.0@9.897 | 3.85192 | -1.846196667 | 9.897 |
| 62 | 190.0@9.8935 | 2.910765333 | -2.002429 | 9.8935 |
| 63 | 205.0@9.895 | 3.625015 | -2.07517 | 9.895 |
| 64 | 205.1@9.895 | 3.625033333 | -2.141915667 | 9.895 |
| 65 | 73.1@11.712999 | -2.3476101 | 2.541779733 | 11.712999 |
| 66 | 73.0@11.712999 | -2.40509 | 2.533589667 | 11.712999 |
| 67 | 56.0@6.994 | 10.66410851 | -0.94812838 | 6.994 |
| 68 | 73.0@26.261332 | 6.914180667 | -3.359300467 | 26.261332 |
| 69 | 361.0@23.788 | 10.51902735 | -0.94812838 | 23.788 |
| 70 | 187.0@12.535167 | 5.006098117 | -0.864216483 | 12.535167 |
| 71 | 187.0@12.5346 | 5.0069037 | -5.646625467 | 12.5346 |
| 72 | 73.0@17.144 | 10.37891501 | -0.94812838 | 17.144 |
| 73 | 73.0@12.5355 | 3.286642833 | -3.297964333 | 12.5355 |
| 74 | 97.4@12.529 | 10.33483435 | -0.94812838 | 12.529 |
| 75 | 72.0@9.8755 | 3.507689467 | -3.218864233 | 9.8755 |
| 76 | 166.9@9.888 | 10.28339785 | -0.94812838 | 9.888 |
| 77 | 239.1@11.8304 | 0.9713046 | -0.4122607 | 11.8304 |
| 78 | 239.1@11.83025 | 1.400039333 | -4.673067667 | 11.83025 |
| 79 | 73.0@8.491 | 10.23141435 | -0.94812838 | 8.491 |
| 80 | 72.1@9.8755 | 3.401405 | -3.218859767 | 9.8755 |
| 81 | 73.1@16.929 | 10.05522443 | -1.041544583 | 16.929 |
| 82 | 73.1@15.908 | 4.716764651 | 3.646897433 | 15.908 |
| 83 | 73.0@15.804 | 4.716764651 | 3.63173829 | 15.804 |
| 84 | 73.1@15.804 | 4.716764651 | 3.609454623 | 15.804 |
| 85 | 73.0@15.899 | 4.716764651 | 3.575837767 | 15.899 |
| 86 | 347.0@16.517 | 4.716764651 | 3.560723433 | 16.517 |
| 87 | 73.1@22.744 | 4.716764651 | 3.534775957 | 22.744 |
| 88 | 73.1@22.738 | 4.716764651 | 3.485525123 | 22.738 |
| 89 | 73.0@16.093 | 4.716764651 | 3.480385623 | 16.093 |
| 90 | 72.0@9.938 | 4.716764651 | 3.433480623 | 9.938 |
| 91 | 465.1@16.519 | 4.716764651 | 3.394428767 | 16.519 |
| 92 | 271.1@23.789 | 9.824833013 | -0.94812838 | 23.789 |
| 93 | 147.0@23.798:2 | 9.799995847 | -0.94812838 | 23.798 |
| 94 | 73.0@18.362 | 9.696329933 | -1.041544583 | 18.362 |
| 95 | 73.1@18.362 | 9.696272433 | -1.041544583 | 18.362 |
| 96 | 73.0@26.274 | 9.773899513 | -0.94812838 | 26.274 |
| 97 | 377.1@16.517 | 9.756477347 | -0.94812838 | 16.517 |
| 98 | 73.0@16.202 | 4.716764651 | 3.314806623 | 16.202 |
| 99 | 73.1@16.202 | 4.716764651 | 3.275262957 | 16.202 |
| 100 | 73.0@22.744 | 4.716764651 | 3.193275623 | 22.744 |
| 101 | 147.0@10.093:1 | 4.716764651 | 3.14528529 | 10.093 |
| 102 | 451.1@23.798 | 9.57028218 | -0.94812838 | 23.798 |
| 103 | 242.0@15.417 | 4.716764651 | 3.1365801 | 15.417 |
| 104 | 73.0@24.705 | 4.716764651 | 3.119164433 | 24.705 |
| 105 | 147.0@10.093:2 | 4.716764651 | 3.117087957 | 10.093 |
