# Supplementary material for: The genotype of barley cultivars influences multiple aspects of their associated microbiota via differential root exudate secretion
Source: PLoS Biol. 2024 Apr 25;22(4):e3002232. doi: 10.1371/journal.pbio.3002232 (PMC11045101; doi:10.1371/journal.pbio.3002232)
Supplement: S3 Table — (DOCX) [file pbio.3002232.s003.docx]

**S3 Table.** Differentially present genes in the *Pseudomonas* populations and their functions.

| **Gene ID** | **Predicted role** |
| --- | --- |
| *PFLU_0242* | Hypothetical protein |
| *PFLU_0315* | Transporters |
| *PFLU_0591* | Siderophores |
| *PFLU_0662* | Transcription factors |
| *PFLU_0704* | Motility |
| *PFLU_0877* | Transporters |
| *PFLU_1022* | Siderophores |
| *PFLU_1028* | Transporters |
| *PFLU_1040* | Siderophores |
| *PFLU_1111* | Signalling |
| *PFLU_1112* | Signalling |
| *PFLU_1260* | Signalling |
| *PFLU_1377* | Genetic information processing |
| *PFLU_1410* | Signalling |
| *PFLU_1433* | Transporters |
| *PFLU_1533* | Transcription factors |
| *PFLU_1629* | Signalling |
| *PFLU_1743* | Signalling |
| *PFLU_1767* | Hypothetical protein |
| *PFLU_1911* | Genetic information processing |
| *PFLU_2157* | Genetic information processing |
| *PFLU_2171* | Hypothetical protein |
| *PFLU_2383* | Transporters |
| *PFLU_2414* | Siderophores |
| *PFLU_2460* | Transcription factors |
| *PFLU_2482* | Hypothetical protein |
| *PFLU_2583* | Transporters |
| *PFLU_2720* | Signalling |
| *PFLU_2764* | Biofilm formation |
| *PFLU_3002* | Hypothetical protein |
| *PFLU_3084* | Hypothetical protein |
| *PFLU_3274* | Transporters |
| *PFLU_3283* | Signalling |
| *PFLU_3295* | Transcription factors |
| *PFLU_3500* | Transporters |
| *PFLU_3566* | Siderophores |
| *PFLU_3620* | Hypothetical protein |
| *PFLU_3633* | Siderophores |
| *PFLU_4219* | Siderophores |
| *PFLU_4387* | Siderophores |
| *PFLU_4512* | Transporters |
| *PFLU_4819* | Motility |
| *PFLU_4886* | Hypothetical protein |
| *PFLU_5080* | Hypothetical protein |
| *PFLU_5308* | Genetic information processing |
| *PFLU_5541* | Hypothetical protein |
| *PFLU_5743* | Signalling |
| *PFLU_5905* | Transporters |
| *PFLU_6047* | Transporters |
| *PFLU_6072* | Transcription factors |
| *PFLU_6101* | Signalling |
