# Supplementary material for: The genotype of barley cultivars influences multiple aspects of their associated microbiota via differential root exudate secretion
Source: PLoS Biol. 2024 Apr 25;22(4):e3002232. doi: 10.1371/journal.pbio.3002232 (PMC11045101; doi:10.1371/journal.pbio.3002232)
Supplement: S5 Table — (DOCX) [file pbio.3002232.s005.docx]

***S5 Table.*** *Bacterial strains used in this work.*

| **Strain** | **Description** | **Reference** |
| --- | --- | --- |
| ***Escherichia coli*** | |  |
| DH5α | *endA*1, *hsdR*17(r_K_-m_K_+), *supE*44, *recA*1, *gyrA* (Nal^r^), *relA*1, Δ(*lacIZYA-argF*)U169, *deoR*, Φ80*dlacΔ(lacZ)M15* | Thermo Fisher Scientific |
| ***Pseudomonas fluorescens*** | |  |
| SBW25 | Environmental *P. fluorescens* isolate | [1] |
| SBW25 ∆*rccR* | *rccR* (*PFLU_6073*) deletion strain | [2] |
| SBW25 ∆*hexR* | *hexR* (*PFLU_4840*) deletion strain | [2] |
| SBW25-*lacZ* | WT SBW25 strain expressing *lacZ* | [2] |
| SBW25 ∆*rccR*-*lacZ* | Mutant *rccR* background expressing *lacZ* | This work |
| SBW25 ∆*gabP* | *gabP* (*PFLU_0315*) deletion strain | This work |
| SBW25 ∆*vanR* | *vanR* (*PFLU_3295*) deletion strain | This work |
| SBW25 ∆*2583* | *PFLU_2583* deletion strain | This work |
| SBW25 ∆*5080* | *PFLU_5080* deletion strain | This work |
| SBW25 ∆*6072* | *PFLU_6072* deletion strain | This work |
| SBW25 ∆*dctA* | *dctA* (*PFLU_3500*) deletion strain | This work |
| SBW25 ∆*1533* | *PFLU_1533* deletion strain | This work |
| SBW25 ∆*2414* | *PFLU_2414* deletion strain | This work |
| SBW25 ∆*3091* | *PFLU_3091* deletion strain | This work |
| SBW25 ∆*4463* | *PFLU_4463* deletion strain | This work |
| Tipple isolates | 120 *Pseudomonas* isolates from the rhizosphere soil of Tipple barley plants | This work |
| Chevallier isolates | 120 *Pseudomonas* isolates from the rhizosphere soil of Chevallier barley plants | This work |
| ***Streptomyces venezuelae*** | |  |
| ATCC 10712 | Laboratory strain | [3] |
| ***R. leguminosarum* bv. viciae 3841 *lux* operon fusions (Rlv3841_*lux*)** | | |
| LMB483 | Phenylalanine biosensor | [4] |
| LMB590 | Xylose biosensor | [4] |
| LMB592 | *myo-*Inositol biosensor | [4] |
| LMB593 | Sucrose biosensor | [4] |
| LMB608 | Tartrate biosensor | [4] |
| LMB610 | Formate biosensor | [4] |
| LMB613 | Salicylic acid biosensor | [4] |
| LMB614 | C4-dicarboxylates biosensor | [4] |
| LMB617 | Mannitol biosensor | [4] |
| LMB619 | Erythritol biosensor | [4] |
| LMB638 | Malonate biosensor | [4] |
| LMB639 | GABA biosensor | [4] |
| LMB667 | Fructose biosensor | [4] |
| OPS0650 | Proline biosensor | [5] |

Reference

1. Rainey PB, Bailey MJ. Physical and genetic map of the Pseudomonas fluorescens SBW25 chromosome. Mol Microbiol. 1996;19(3):521-33. Epub 1996/02/01. PubMed PMID: 8830243.

2. Campilongo R, Fung RKY, Little RH, Grenga L, Trampari E, Pepe S, et al. One ligand, two regulators and three binding sites: How KDPG controls primary carbon metabolism in Pseudomonas. PLoS Genet. 2017;13(6):e1006839. Epub 2017/06/29. doi: 10.1371/journal.pgen.1006839. PubMed PMID: 28658302; PubMed Central PMCID: PMCPMC5489143.

3. Bibb MJ, Domonkos A, Chandra G, Buttner MJ. Expression of the chaplin and rodlin hydrophobic sheath proteins in Streptomyces venezuelae is controlled by sigma(BldN) and a cognate anti-sigma factor, RsbN. Mol Microbiol. 2012;84(6):1033-49. Epub 2012/05/16. doi: 10.1111/j.1365-2958.2012.08070.x. PubMed PMID: 22582857.

4. Pini F, East AK, Appia-Ayme C, Tomek J, Karunakaran R, Mendoza-Suarez M, et al. Bacterial Biosensors for in Vivo Spatiotemporal Mapping of Root Secretion. Plant physiology. 2017;174(3):1289-306. Epub 2017/05/13. doi: 10.1104/pp.16.01302. PubMed PMID: 28495892; PubMed Central PMCID: PMCPMC5490882.

5. Rubia MI, Ramachandran VK, Arrese-Igor C, Larrainzar E, Poole PS. A novel biosensor to monitor proline in pea root exudates and nodules under osmotic stress and recovery. Plant and Soil. 2020;452(1):413-22. doi: 10.1007/s11104-020-04577-2.
