# Supplementary material for: The genotype of barley cultivars influences multiple aspects of their associated microbiota via differential root exudate secretion
Source: PLoS Biol. 2024 Apr 25;22(4):e3002232. doi: 10.1371/journal.pbio.3002232 (PMC11045101; doi:10.1371/journal.pbio.3002232)
Supplement: S6 Table — (DOCX) [file pbio.3002232.s006.docx]

**S6 Table.** Plasmids used in this study.

| **Plasmid** | **Description** | **Antibiotic Resistance** | **Reference** |
| --- | --- | --- | --- |
| pTS1 | Suicide plasmid, pME3087 derivative, *sacB* | Tet | [1] |
| pME3087_∆*6073* | pME3087 containing 500 bp flanking regions of *PFLU_6073* | Tet | [2] |
| pTS1_∆*PFLU0315* | pTS1 based synthetic construct for deletion of *PFLU_0315* | Tet | This work |
| pTS1_∆*PFLU3295* | pTS1 based synthetic construct for deletion of *PFLU_3295* | Tet | This work |
| pTS1_∆*PFLU5080* | pTS1 based synthetic construct for deletion of *PFLU_5080* | Tet | This work |
| pTS1_∆*PFLU6072* | pTS1 based synthetic construct for deletion of *PFLU_6072* | Tet | This work |
| pTS1_∆*PFLU3500* | pTS1 based synthetic construct for deletion of *PFLU_3500* | Tet | This work |
| pTS1_∆*PFLU1533* | pTS1 based synthetic construct for deletion of *PFLU_1533* | Tet | This work |
| pTS1_∆*PFLU2414* | pTS1 based synthetic construct for deletion of *PFLU_2414* | Tet | This work |

Reference

1. Scott TA, Heine D, Qin Z, Wilkinson B. An L-threonine transaldolase is required for L-threo-β-hydroxy-α-amino acid assembly during obafluorin biosynthesis. Nature communications. 2017;8(1):1-11.

2. Campilongo R, Fung RKY, Little RH, Grenga L, Trampari E, Pepe S, et al. One ligand, two regulators and three binding sites: How KDPG controls primary carbon metabolism in Pseudomonas. PLoS Genet. 2017;13(6):e1006839. Epub 2017/06/29. doi: 10.1371/journal.pgen.1006839. PubMed PMID: 28658302; PubMed Central PMCID: PMCPMC5489143.
