# Supplementary material for: The genotype of barley cultivars influences multiple aspects of their associated microbiota via differential root exudate secretion
Source: PLoS Biol. 2024 Apr 25;22(4):e3002232. doi: 10.1371/journal.pbio.3002232 (PMC11045101; doi:10.1371/journal.pbio.3002232)
Supplement: S7 Table — (DOCX) [file pbio.3002232.s007.docx]

**S7 Table.** Oligonucleotides used in this work.

| **Name** | **Sequence (3'-5')** | **Description** |
| --- | --- | --- |
| gyrB-UP1 | CAYGCNGGNGGNAARTTYGA | To amplify *gyrB* as in [1] |
| gyrB-UP2R | CCRTCNACRTCNGCRTCNGTCAT | To amplify *gyrB* as in [1] |
| PFlu6073UPOUT | CGGTGCACCAACTGCGTCAA | To confirm *PFLU_6073* deletion |
| PFlu6073DNOUT | AACGCGTGCACTTCACCCAG | To confirm *PFLU_6073* deletion |
| KOPFLU3500_F | TCTTGGCCCGATTGCGCA | To confirm *PFLU_3500* deletion |
| KOPFLU3500_R | CTTCGCGCATGTCCATCA | To confirm *PFLU_3500* deletion |
| KOPFLU0315_F | TCATTGCGATCTTCGCTG | To confirm *PFLU_0315* deletion |
| KOPFLU0315_R | ATGGCCTCGCCGAACAAG | To confirm *PFLU_0315* deletion |
| KOPFLU3295_F | AAGACTGTCTGCACCGCG | To confirm *PFLU_3295* deletion |
| KOPFLU3295_R | TGCGTGACAGCCGTATCG | To confirm *PFLU_3295* deletion |
| KOPFLU5080_F | GGAGCAAAGAACCCAACC | To confirm *PFLU_5080* deletion |
| KOPFLU5080_R | TGAGCCGCTTTGTTCTCG | To confirm *PFLU_5080* deletion |
| KOPFLU6072_F | CCATGCGGCGGATGACTT | To confirm *PFLU_6072* deletion |
| KOPFLU6072_R | GTGCATGAAGACACCGAG | To confirm *PFLU_6072* deletion |
| KOPFLU1533_F | GTTTGAAACCACCGCGCG | To confirm *PFLU_1533* deletion |
| KOPFLU1533_R | GGGTTGGCCATCATGTCG | To confirm *PFLU_1533* deletion |
| KOPFLU2414_F | TTCAAGGTGCGTTTGCGC | To confirm *PFLU_2414* deletion |
| KOPFLU2414_R | ATCATGCTCATGGCGGTG | To confirm *PFLU_2414* deletion |
| up700_2583_F | ATTCGAGCTCGGTACCCGGGCCGCGTTTCAGGGTCAGTG | SBW25 deletion of *PFLU_2583*, for Gibson assembly into pTS1 |
| up700_2583_R | GCTACTTGAAACGGATCGGGGTCTTCATG | SBW25 deletion of *PFLU_2583*, for Gibson assembly into pTS1 |
| down700_2583_F | CCCGATCCGTTTCAAGTAGCCCTGACATAAC | SBW25 deletion of *PFLU_2583*, for Gibson assembly into pTS1 |
| down700_2583_R | CCTGCAGGTCGACTCTAGAGGACGATGCCTTTGCCCTG | SBW25 deletion of *PFLU_2583,* for Gibson assembly into pTS1 |
| KO2583_mutantcheckF | CTTGCAAAACAGCCAGGCCC | To confirm *PFLU_2583* deletion |
| KO2583_mutantcheckR | ATGGCAAACACTTCGTTCATTTT | To confirm *PFLU_2583* deletion |
| up3091_F | ATCGATCCGAATTCGAGCTCGGTACCCGGGAAAACGCTCCACCTGTTGG | SBW25 deletion of *PFLU_3091*, for Gibson assembly into pTS1 |
| up3091_R | GGAGCCTTAAAGCTTCATCGGCACCGGTTAGCG | SBW25 deletion of *PFLU_3091*, for Gibson assembly into pTS1 |
| down3091_F | TAACCGGTGCCGATGAAGCTTTAAGGCTCCAGC | SBW25 deletion of *PFLU_3091*, for Gibson assembly into pTS1 |
| down3091_R | AGCTTGCATGCCTGCAGGTCGACTCTAGAGCAAGGGTTTGCGGTGTAG | SBW25 deletion of *PFLU_3091,* for Gibson assembly into pTS1 |
| up4463_F | ATCGATCCGAATTCGAGCTCGGTACCCGGGGACCCGCTGATCGCCCAG | SBW25 deletion of *PFLU_4463*, for Gibson assembly into pTS1 |
| up4463_R | TGTAGATCACGCCGTGGGAGTCATTGCAGTCCTTG | SBW25 deletion of *PFLU_4463,* for Gibson assembly into pTS1 |
| down4463_F | ACTGCAATGACTCCCACGGCGTGATCTACACTTTAAAC | SBW25 deletion of *PFLU_4463*, for Gibson assembly into pTS1 |
| down4463_R | AGCTTGCATGCCTGCAGGTCGACTCTAGAGGACCCGCAGCGTGACCTG | SBW25 deletion of *PFLU_4463,* for Gibson assembly into pTS1 |
| KO4463_test_F | CAGTCGCCGCCTGCTCGA | To confirm *PFLU_4463* deletion |
| KO4463_test_R | ATCTGGGAGCGTAAGGCCG | To confirm *PFLU_4463* deletion |
| KO3091_test_F | GGCAATCATGAACGGCCAGT | To confirm *PFLU_3091* deletion |
| KO3091_test_R | AAGCCTTCGCCATACAGTGC | To confirm *PFLU_3091* deletion |

Reference

1. Yamamoto S, Harayama S. PCR amplification and direct sequencing of gyrB genes with universal primers and their application to the detection and taxonomic analysis of Pseudomonas putida strains. Applied and Environmental Microbiology. 1995;61(3):1104-9. doi: doi:10.1128/aem.61.3.1104-1109.1995.
