# Supplementary figures and images for: The genotype of barley cultivars influences multiple aspects of their associated microbiota via differential root exudate secretion
Source: PLoS Biol. 2024 Apr 25;22(4):e3002232. doi: 10.1371/journal.pbio.3002232 (PMC11045101; doi:10.1371/journal.pbio.3002232)

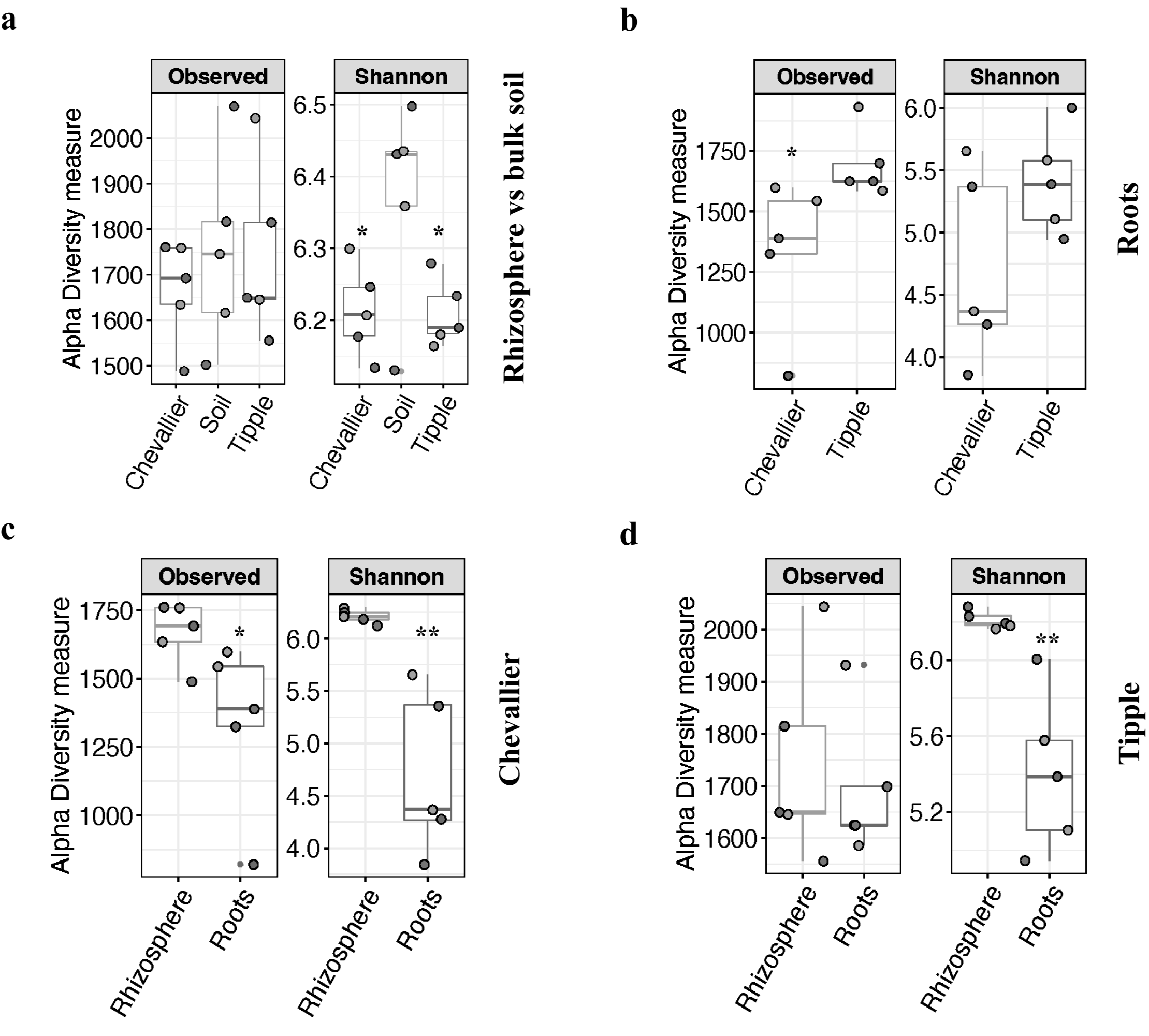

Supplement: S1 Fig — Observed richness and Shannon diversity were used as diversity measures. (A) Overall comparison between the rhizosphere communities of Chevallier and Tipple and the bulk soil. (B) Root endosphere community comparison between Chevallier and Tipple. (C) Comparison of community composition between Chevallier compartments. (D) Comparison of community composition between Tipple compartments. Five replicates, represented as different coloured dots, were used per condition. Asterisks indicate p < 0.05 (*), 0.01 (**), or 0.001(***). The data underlying this figure can be found in S8 Data. (TIF) [file pbio.3002232.s008.tif]

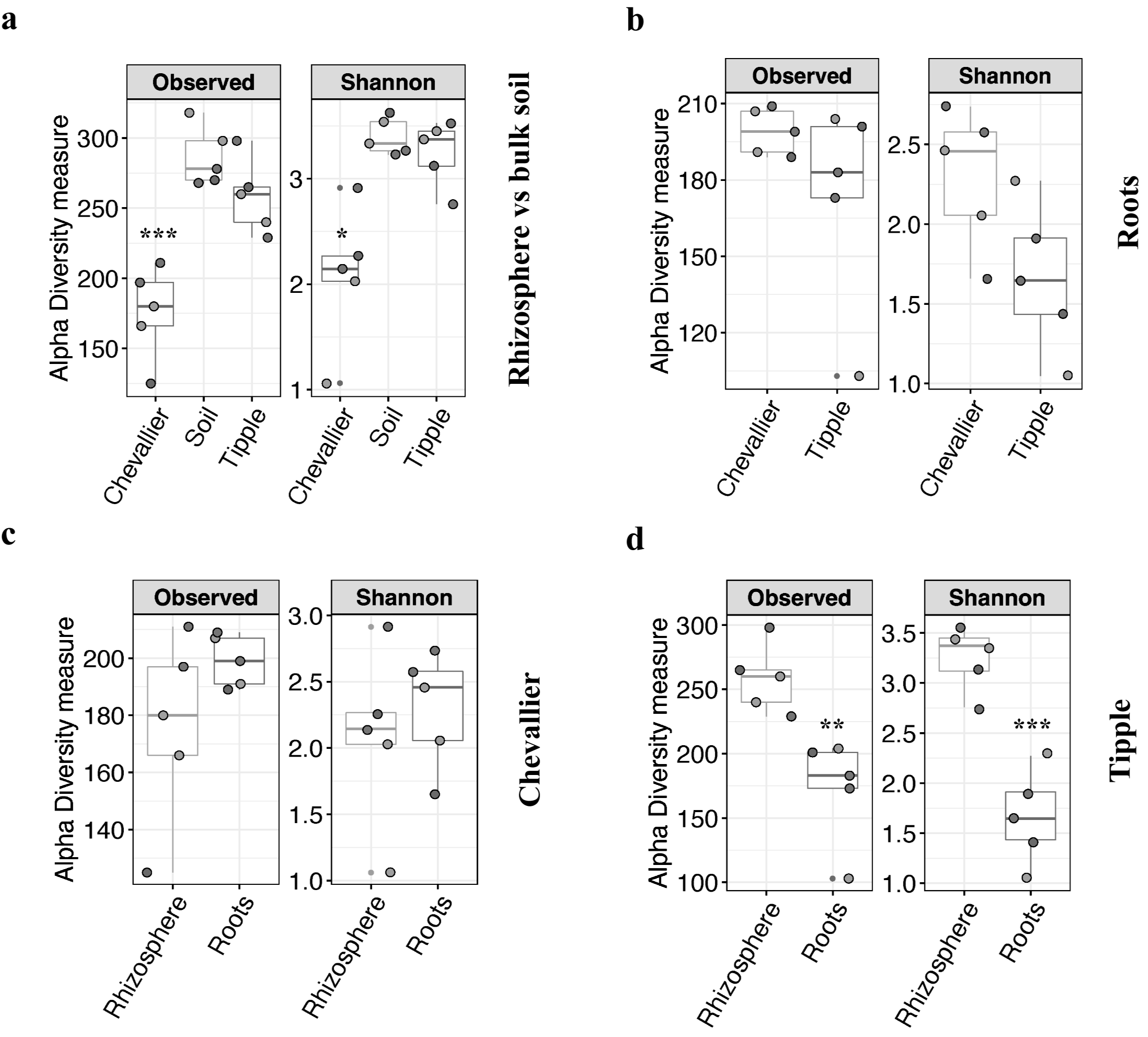

Supplement: S2 Fig — Observed richness and Shannon diversity were used as diversity measures. (A) Overall comparison between the rhizosphere communities of Chevallier and Tipple and the bulk soil. Significant differences for both indices, between cultivars and Chevallier and bulk soil. (B) Root endosphere community comparison between Chevallier and Tipple. No significant differences were found. (C) Comparison of community composition between Chevallier compartments. (D) Comparison of community composition between Tipple compartments. Five replicates, represented as different coloured dots, were used per condition. Asterisks indicate p < 0.05 (*), 0.01 (**), or 0.001(***). The data underlying this figure can be found in S9 Data. (TIF) [file pbio.3002232.s009.tif]

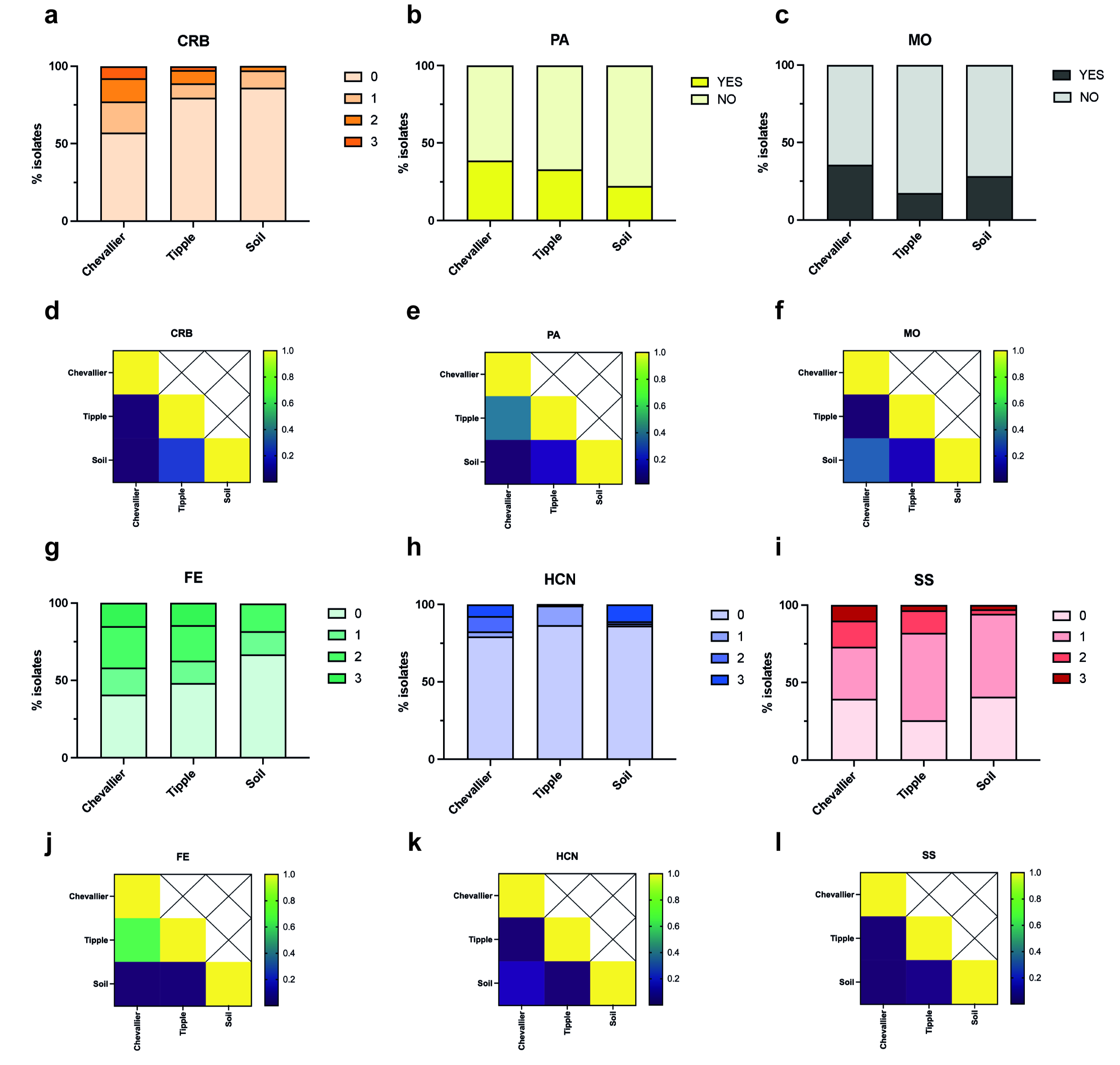

Supplement: S3 Fig — (A) Congo red binding (CRB). (B) Protease activity (PA). (C) Motility (MO). (D) CRB Chi-Square paired comparisons. (E) PA Chi-Square paired comparisons. (F) MO Chi-Square paired comparisons. (G) Fluorescence emission (FE). (H) Hydrogen cyanide production (HCN). (I) Streptomyces suppression (SS). (J) FE Chi-Square paired comparisons. (K) HCN Chi-Square paired comparisons. (L) SS Chi-Square paired comparisons. Data is shown as the relative percentage of isolates presenting a given score. Significant differences according to Chi-square test are represented. The data underlying this figure can be found in S10 Data. (TIF) [file pbio.3002232.s010.tif]

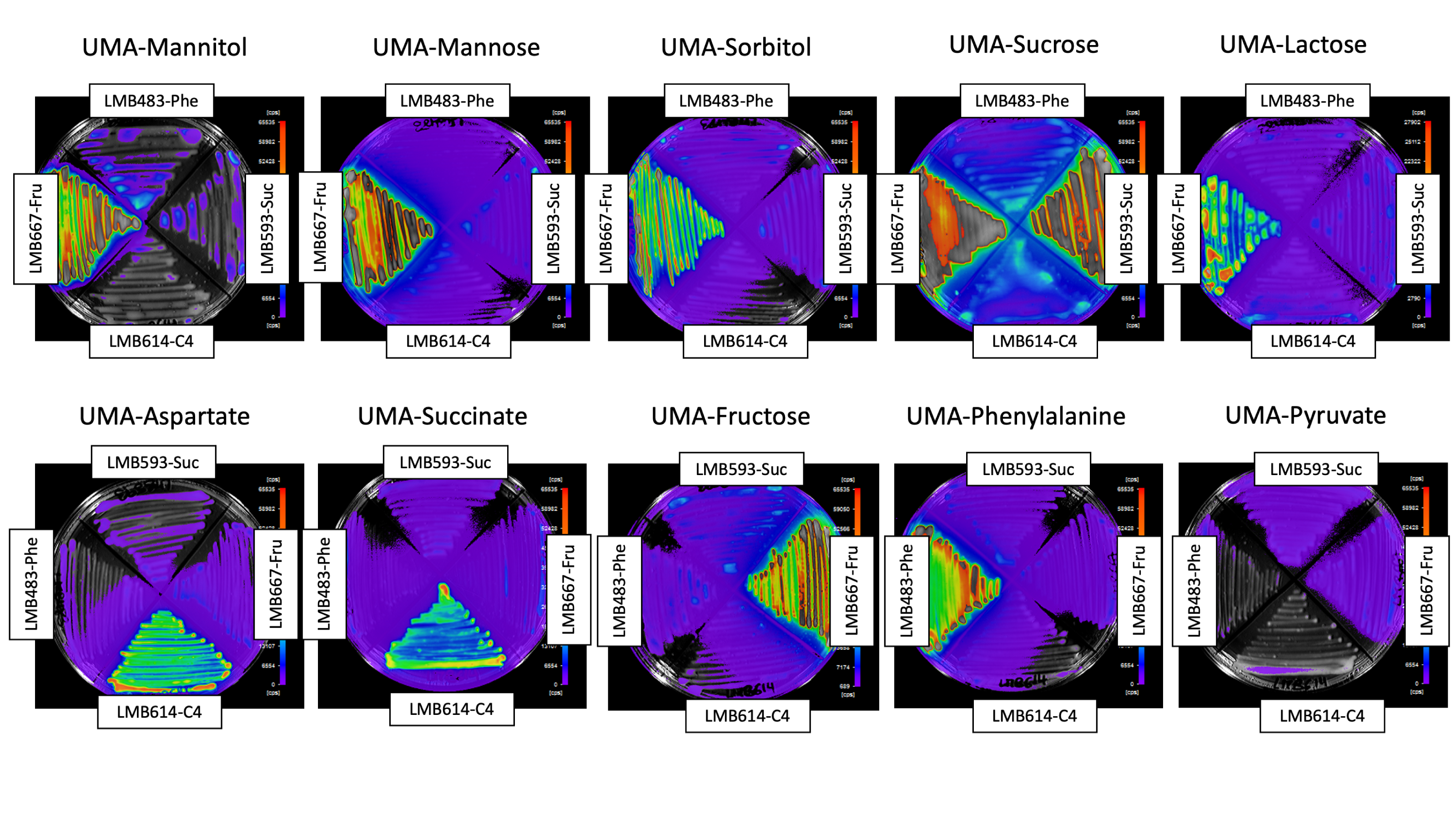

Supplement: S4 Fig — Aspartate, succinate, and phenylalanine plates were additionally supplemented with 30 mM pyruvate to enable bacterial growth. Biosensors used are listed in S5 Table. (TIF) [file pbio.3002232.s011.tif]

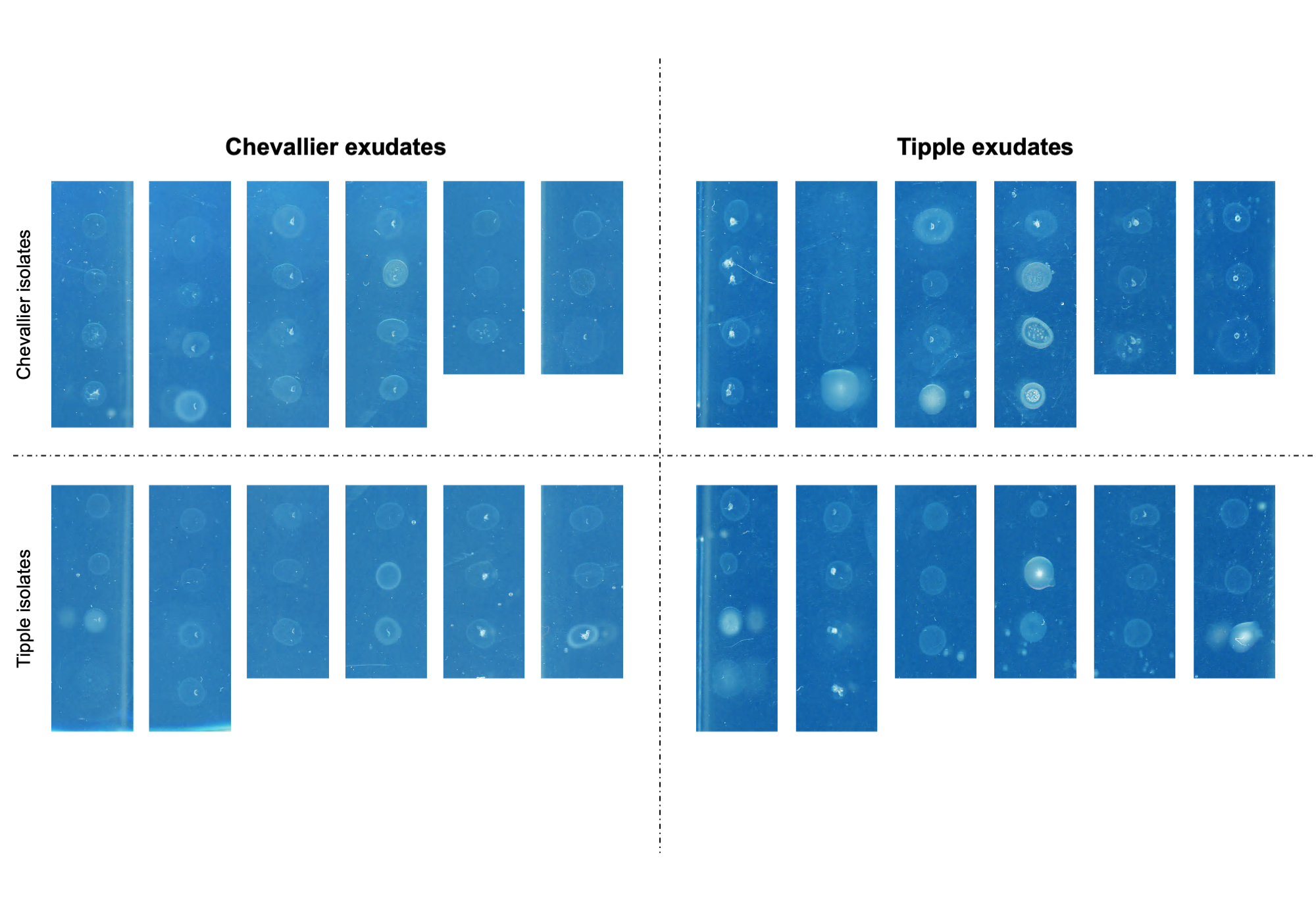

Supplement: S5 Fig — (TIFF) [file pbio.3002232.s012.tiff]

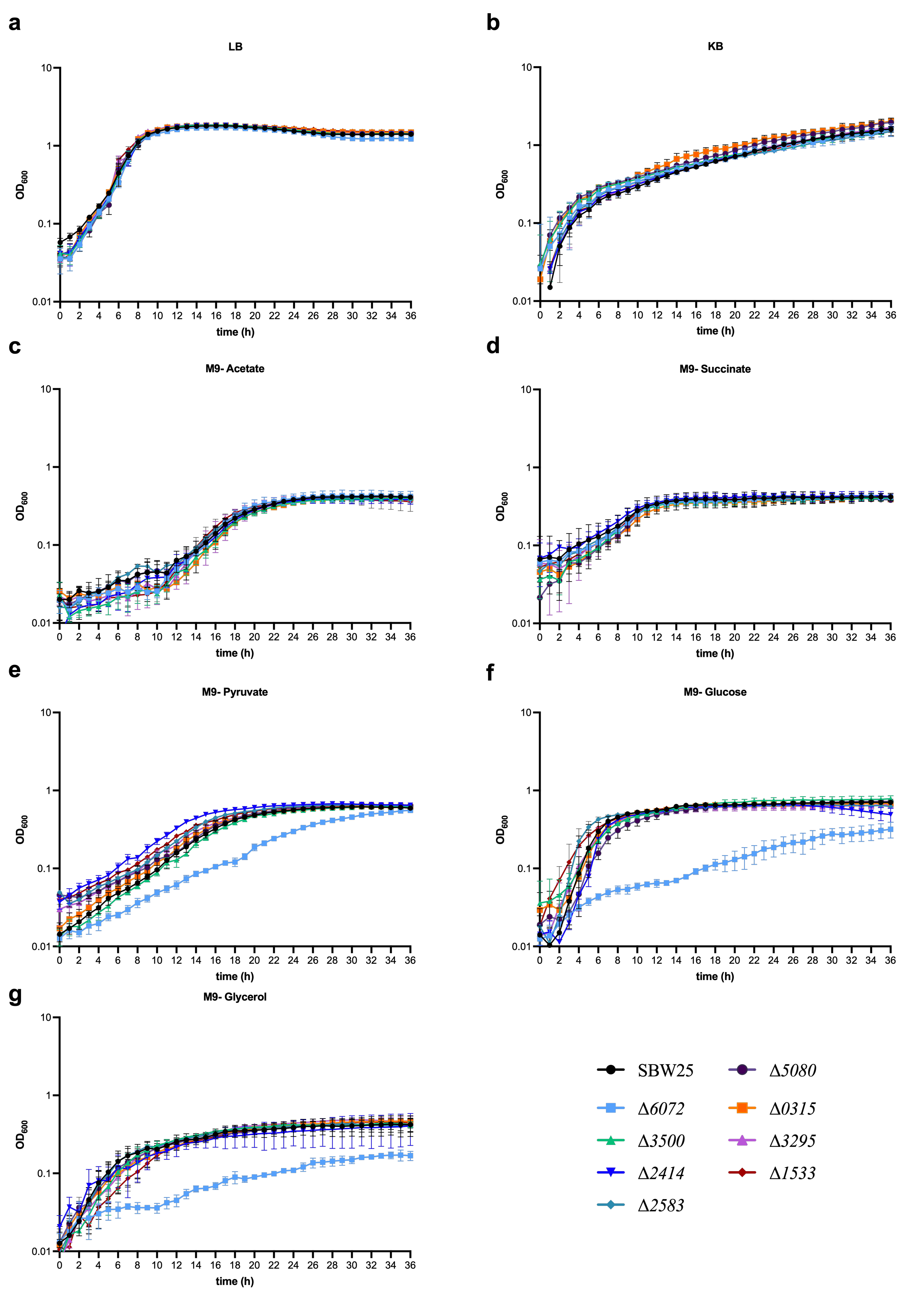

Supplement: S6 Fig — (A) LB medium. (B) KB medium. (C) M9 minimal medium 0.4% acetate. (D) M9 minimal medium 0.4% succinate. (E) M9 minimal medium 0.4% pyruvate. (F) M9 minimal medium 0.4% glucose. (G) M9 minimal medium 0.4% Glycerol. Three biological reps used per strain. Error bars are represented as SEM. Experiment was repeated 3 times and here a representative graph is shown. The data underlying this figure can be found in S11 Data. (TIFF) [file pbio.3002232.s013.tiff]

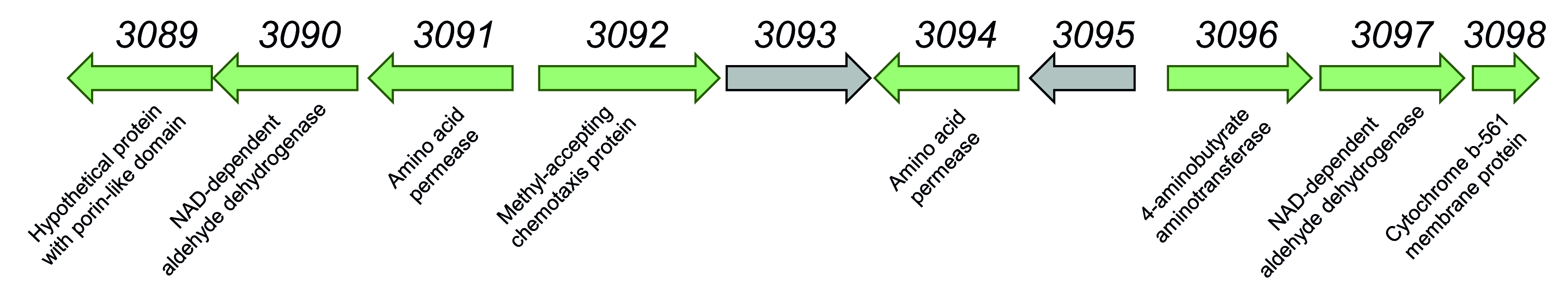

Supplement: S7 Fig — Gene numbers are indicated above each gene. Genes up-regulated in the Tipple rhizosphere relative to Chevallier are highlighted in green. Predicted encoded protein functions are given for up-regulated genes. Arrows indicate the direction of open reading frame transcription in each case. (TIF) [file pbio.3002232.s014.tif]

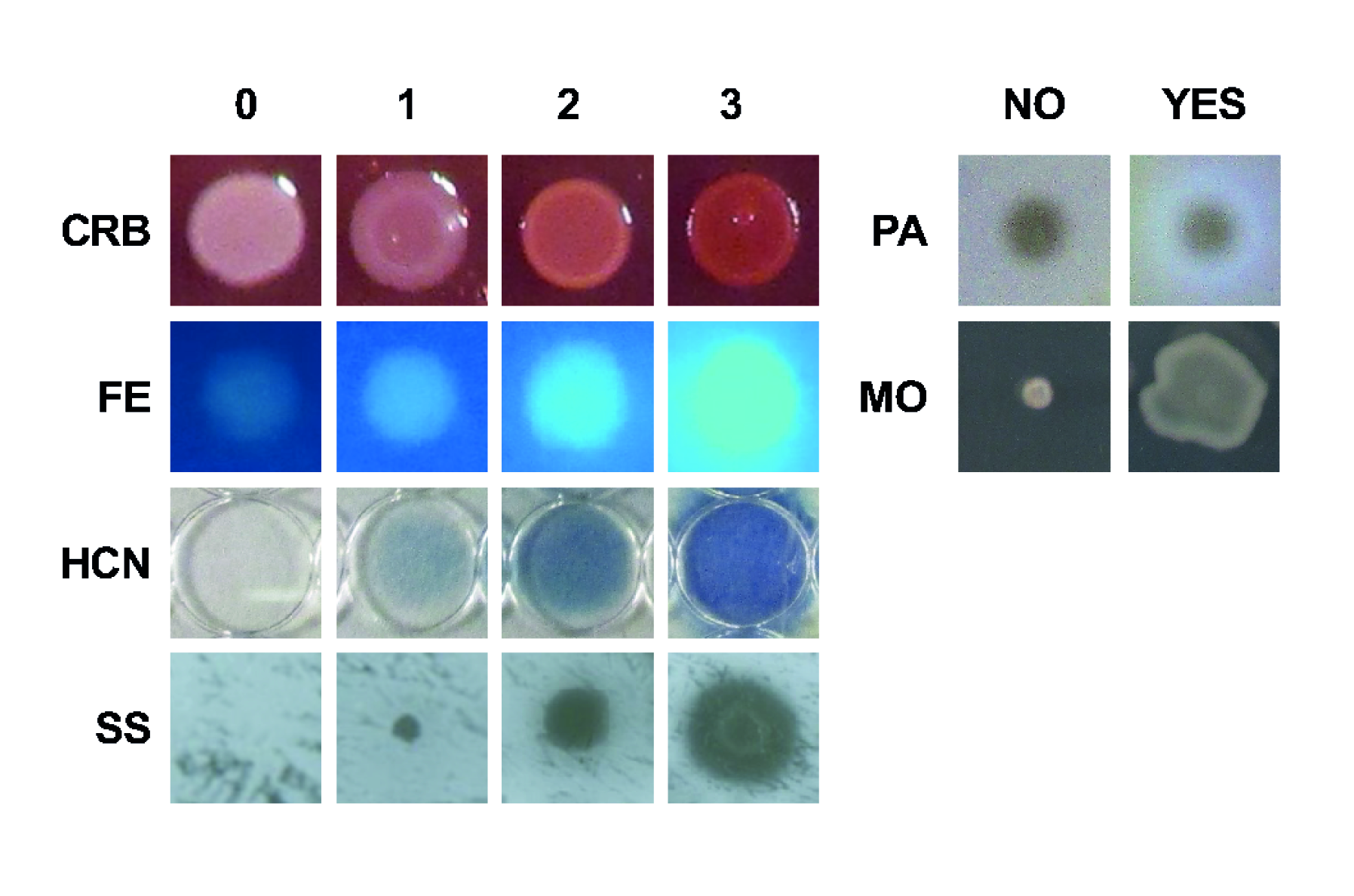

Supplement: S8 Fig — (TIF) [file pbio.3002232.s015.tif]
