# Supplementary material for: The genotype of barley cultivars influences multiple aspects of their associated microbiota via differential root exudate secretion
Source: PLoS Biol. 2024 Apr 25;22(4):e3002232. doi: 10.1371/journal.pbio.3002232 (PMC11045101; doi:10.1371/journal.pbio.3002232)
Supplement: Attachment — Submitted filename: Response to Reviewers.docx [file pbio.3002232.s027.docx]

Reviewer #1: In this elegant study, Pacheco-Moreno et al. study the microbiomes different barley cultivars assemble and aim to identify how Pseudomonas adapt to their host cultivar, the role of selected genes in rhizosphere competence and role of cultivar-specific microbiomes in plant performance. The study employs a range of well executed experiments and reaches some well-proven conclusions. Nevertheless, I have a number of comments that can be found below.

We thank both reviewers for their detailed and positive assessments of our study, and attempt to fully address their comments in the responses below.

Major comments

1.	In the figure preparation, I would prefer the bulk soil on the left side of the graph followed by the root or rhizosphere samples. Please take this into account in all figures about microbiome analysis.

We have amended Figure 1 to include the bulk soil on the left as requested.

2.	Lines 208-217, what about fructose that displayed highest difference in the previous assay (luminescent biosensor)?

We could not detect fructose in our exudate samples. To address the discrepancy, we examined the range of sugars that would trigger the fructose biosensor used in our assays. We were able to confirm the previous work by Pini et al. (2017), who showed that the fructose biosensor responds to mannitol, mannose, sorbitol and lactose in addition to fructose. We also tested whether the fructose biosensor also responded to glucose but it did not, at least in our plate assays. We have added these tests to the supplementary material (Figure S4) and amended the text to make clear that the results for the fructose biosensor may indicate increased abundance of one or more hexose sugar molecules.

3.	Lines 230-239, in previous experiment, axenic exudates were collected from the 2 cultivars and around 100 entities were found as differentially abundant. Did the authors consider observing growth of selected isolates on the "total" exudates, instead of testing specific carbon sources? I feel that it would be an extra confirmation about preferential growth of isolates on host-specific exudates.

This is a good suggestion, which we considered as part of our initial analysis of the isolated *Pseudomonas* strains. However, the volume of root exudates required to conduct growth curves for our isolate library would have required very large numbers of plants and substantial time and effort to produce. Given the effort required to produce enough sample for robust GC/MS analysis, and considering the constraints of researcher time and funding, this experiment was difficult to justify.

4.	Lines 309-323, considering that you generated a set of mutants adapted in each rhizosphere, it would be nice to also perform some growth assays with SBW25 and compare its effect to the mutants when they are applied in each cultivar. That will also show whether bacterial genes are not only linked with colonization but also with downstream effects such as growth.

This is an interesting idea; however we feel it is unlikely to produce insightful results for a couple of reasons. First is the inherently noisy nature of our microbiome swap data. Given that the growth impacts we saw upon inoculation with barley-selected *Pseudomonas* communities in Figure 7 were smaller than were seen for whole rhizosphere microbiomes, we would expect any impact from individual gene deletions to be smaller still, almost certainly falling within the margin of error for growth promotion. Secondly SBW25, the model organism used in our competition assays is not a barley isolate, originally coming from sugar beet. Any growth promotion effects seen with this organism would therefore be non-specific and unlikely to produce distinct, cultivar-specific responses.

Minor comments

1.	Lines 115-123, please mention the figure that you are describing.

This refers to Figure 1a. We have amended the text as requested.

2.	Lines 179-180, it would be nice to have info on isolates in a suppl. table.

A supplementary table (Supplementary Table 1) with this strain information has been added to the manuscript.

3.	Lines 194-198, it would benefit the reader to have this percentage in numbers in the text such as 13/45 isolates displayed trait x.

We have amended the text as requested.

4.	Line 226, 7/60 cannot be considered as several but as "only a few".

We have amended this section of the text to make it more precise, as stated below.

5.	In Figure 4, good to add a dashed line at OD=0.3 since now the focus is on the dashed line that is lower than 0.3.

With hindsight our presentation of these data was a little confusing, and we apologise for this. We have changed this figure and the corresponding text (lines 226-229) to show strains that grow to a higher OD600 than the 90^th^ percentile value for each assay. These values are 0.2796 for Acetate and 0.2028 for Glucose. This does not change our interpretation of the data, but does give more precise description of what we see here.

6.	Figure 4e has low resolution. Replace with a higher quality one.

We have replaced the panel with a higher resolution version as requested.

7.	Figure 5a, it would be informative if the genes can be organized based on function and there is some info on the graph about general role (e.g. transporters, signaling, etc).

This is a great suggestion. Unfortunately when we attempted to revise the figure, it looked very crowded and was difficult to interpret. Therefore we included the relevant information in Supplementary Table 3.

8.	Lines 261-264, this is not what is shown in figure 5b.

The text on lines 261-264 refers to an earlier version of the figure which included this data. We clearly missed this during the drafting period, and apologise for the confusion. As Supplementary table 2 now gives additional information on gene function, we have deleted this sentence.

9.	Line 479, is it indeed 500 cm2 square plates?

Yes, this is correct. We have amended the text to make this clear.

10.	In Materials and Methods, it should be Petri and not petri. Please also remove space between number and Celsius when mentioning temperatures (e.g. 4oC).

The text has been corrected throughout.

11.	Line 701, it should be suppl. figure 5 and not 3. Please check all figure numbers in the text for correctness.

Supplementary figure numbering has been corrected throughout.

Reviewer #2:

In this manuscript Pacheco-Moreno et al. present evidence that two barley cultivars (one modern and one heirloom cultivar) exhibit differences in root exudate quality, which preferentially select different microbiomes & particularly different subsets of *Pseudomonas fluorescens* to their rhizospheres; and further, that the respective subsets of *P. fluorescens* show higher relative fitness on their preferred type of root exudates; that the differences in root exudate profiles are sufficient to alter gene expression of a reference strain of *P. fluorescens*; and finally that the subsets of *P. fluorescens* preferentially improve growth of the cultivar whose root exudates they prefer.

As a whole, if all of these conclusions are justified, then this would be perhaps the most complete demonstration (that I am aware of) of the causes and consequences of genetically-induced microbiome variation in a major crop plant. It would be remarkable to see a single piece of work that demonstrates an effect of host genotype on microbiome content, identifies the underlying trait, and confirms reciprocal fitness benefits for both host and microbe, as this manuscript proposes. So to my mind, the potential impact of the work is clearly high.

However, I have some questions and concerns about the strength of evidence for some of these claims.

All of the data came from experiments done in the lab or growth chamber. This approach has a lot of benefits, many of which are exemplified by this paper, but it comes with a fairly major drawback: a lack of generalizability to real-life field scenarios. This is not just an academic complaint- the difficulty of reproducing lab results in the field has been one of the primary obstacles preventing the deployment of very promising synthetic microbial communities for agricultural benefit. Now, I do appreciate the value of reductionist work like this, but I think it is important to address the downsides. The authors need to be up front about the setting of the work throughout the paper - i.e. the reader should not be left wondering until the methods section whether this was a field study. The authors also need to revise the Discussion to address the (extremely likely) possibility that host-microbiome dynamics are not so neat and tidy in the field.

We absolutely agree with the reviewer that translating lab studies into real-world field conditions can be extremely difficult, and that lab studies generally have this inherent drawback. We have revised the manuscript in several places including the discussion (e.g. see lines 436-444), to make clear that this is a laboratory study and that reproducing these results in a field setting is a key challenge for the future.

As part of this, there should be explicit discussion of how the presence of other, live microbes could modify the behavior and relative fitnesses of the cultivar-specific P. fluorescens strains. The Methods section indicate that even the most realistic part of the study — the amplicon sequencing data in the first section of the Results—came from plants grown in quite artificial conditions, i.e. a peat-based soilless potting media, which has been shown to harbor a microbiome that is largely unrepresentative of what is found in real soils. On that note I found it very odd that the authors chose to autoclave the field soil before mixing it with the potting media- without autoclaving, it could have been a rich inoculum of real soil microbiome. So in summary, while I enjoyed reading this paper and the story it told, I am skeptical that its main conclusions can be generalized to plant-microbe interactions in the real world.

The soil in JIC Cereal Mix potting compost is routinely sterilised to kill soil-borne cereal pathogens such as *Gaeumannomyces graminis* (take-all). While we personally would not have sterilised the soil in our compost for the reasons stated by the reviewer, given the resources available to us and the fact that our experiments were planned to take place over an extended period of time, we ultimately decided to use JIC Cereal Mix as a consistent and readily available growth medium for all plant studies in complex microbiome settings.

We have amended the text (e.g. lines 104-107, line 440) to make clear that the microbiome in our experiments is likely to differ from that found in many agricultural soils. Nonetheless, it is important to note that this compost still contains a rich and diverse soil microbiome, and the basic principles of exudate-driven microbiome shaping that we describe here are likely to occur on similar lines in any complex soil community.

The statistical support for some of the claims was questionable. In some cases, I simply did not understand the logic behind the choices of statistical tests. For example, in the first section of the Results, some microbiome comparisons used ANOSIM while others used ADONIS, with no reason given for the frequent switching between methods. Both are valid permutational MANOVA methods (though ADONIS is more robust), but why not just pick one and use it? Another unusual switching between methods is found in lines 158-164, where a Tukey post-hoc method was used for one alpha diversity metric and a very different approach - pairwise Wilcoxon test - was used for another metric. It must not be a matter of needing a non-parametric test due to problems meeting the assumptions for ANOVA, because in line 164 the ANOVA is used for both. Even if that were the justification, the Wilcoxon test is not an appropriate substitute for ANOVA followed by Tukey HSD: first, it can only be used to compare two groups whereas ANOVA can handle multiple groups; second, it should only be used on paired data, which (as far as I can tell) this experiment did not have. If there was some sort of pairing mechanism, it needs to be explained in the Methods.

The rationale for our statistical treatment of the different pairs is now explained more fully on lines 573-581 (for Alpha diversity) and 585-588 (for Beta diversity).

In other cases, the statistical approach lacked sufficient detail. For example, simply reporting the p-value for an ANOVA tells me nothing about whether any particular explanatory variable was associated with the response variable (microbiome). To interpret ANOVA results, it is mandatory to specify exactly what explanatory variables, covariates, interactions, etc. were included in the model, as well as what method of calculating sums of squares was used (Type I, Type II, etc.). Different variables each have their own F statistic, df and p-value, which should be reported in the text as support instead of a p-value for the ANOVA in general. In simple cases (one-way ANOVA) they may be the same but as written the reader has no way to know that.

Additional information describing the Anova statistics is now included in the manuscript and can be found on lines 156-163.

Finally, some claims had no statistical support at all, for example many of the comparisons of individual genera between cultivars or between compartments (lines 133-142, e.g.).

The data for comparison of the individual genera is based on the beta-diversity analyses shown in Figure1C and noted in the text on lines 112-113 (bacterial) and 141-142 (fungal). However, the statements we make about the relative abundances of individual genera are qualitative. We have reviewed the text and made a few changes to clarify this section.

Another example is the GC-MS data, and another is lines 215-220 where no evidence is given that the differences between Tipple and Chevallier exudates are strong enough to rule out whether it was just noise.

Statistical support for the genotype effect in the GC/MS data has been added to the text (lines 213-215).

In summary, at least some of the data will need to be re-analyzed using appropriate statistical methods, and in general the Methods section should be expanded to provide adequate detail about what analyses were conducted. The authors should also be careful not to over-interpret their results when the statistical support is actually weak (for example, claims about the benefits of cultivar-selected microbiomes on plant growth).

As stated above, we have revised the manuscript in several places, including the Materials and Methods and Results to provide additional detail on the statistics used, and to address the reviewer’s concerns relating to interpretation of our results.

Minor comments.

Line 67-68: I would not consider nodule formation to be a good representation of genotype effects on the rhizosphere more generally - nodulation is such a specific tightly defined interaction, whereas interactions in the rhizosphere are much more diffuse.

We thought it was worth including this reference to include examples form as broad a cross-section of the plant-microbe interaction literature as possible. However, we take the reviewer’s point here and have removed this sentence from the introduction.

Lines 87-106: This is a long description of the results which I do not think belong in the introduction section.

We have amended the introduction to shorten this section, and moved relevant text to the results.

Line 127: "Bacterial composition between cultivars" - is this referring to all 3 compartments together?

Yes, this refers to the whole plant in each case, and this has been added to the text.

Figure 4: I find this figure kind of hard to interpret - is this really the clearest way to show these data? Why not just show the 48h timepoint or the maximum for each isolate?

We respectfully disagree with the reviewer here. Showing the 48 hour/maximum timepoint only would result in a chart with the same heights of each point, but missing any information about the rate of growth over time for the different isolates. As there are too many growth curves here to reasonably present conventionally, we would like to keep the (revised, see response to Reviewer 1) figure as it is.

Figure 2: Please write out the abbreviations in "Isolate phenotypes"

This has been included in the figure legend for simplicity of the figure.

The caption for Figure 3b states that the heatmap shows metabolites with "a log2-fold change >= 2 in at least one cultivar". This is confusing to me, what is the log2 fold change in comparison to? Why not test for a log2 fold change between the cultivars?

This is indeed a log 2-fold change between the cultivars. We have changed the figure legend for clarity.

Line 681: How much did the volumes need to be adjusted? Was the dilution the same for both rhizosphere suspensions?

4 plants/cultivar were used to prepare the rhizosphere suspension and the dilution was the same for both. The final washed pellet in each cultivar was resuspended into 200 ml PBS from which 5 ml were used for the inoculations. This has been clarified in the text on line 710.

Line 646: Does this mean that 12 plants went in to each rhizosphere sample, or were 12 plants used total?

12 plants went into each sample. We have amended the text to clarify this.

Lines 647-649: So each individual plant went into 20 mL liquid, or did multiple plants combine in one tube? Also, this is the first mention of "sample tubes containing vermiculite"

Each plant tube received 20 mL of liquid, and these samples were then combined and filtered. We have clarified this in the text.

Methods section: Maybe I missed it but it seems that the "rooting solution" mentioned several times is never defined… what was in it?

The recipe for rooting solution is listed in the section *Root colonisation assays* on lines 596-598 of the original manuscript (now lines 625-627).

Metabarcoding study: How did the authors control for variation among samples in sequencing depth, which is known to be a very powerful driver of microbiome profiles and a potential confounding variable?

This issue was considered during the design and conduct of our wet-lab experiments. Following gDNA extraction we conducted a quality control step with the same input material in all cases. We then only shipped successful samples with adequate yields for sequencing. We advised the sequencing provider to only supply data of similar sequencing depth. This worked very well, and we obtained libraries counting an average of 151,631 reads and ranging from 132,836 to 169,149 reads. As we did not observe any libraries to fail or deliver low amounts of reads, we refrained from rarefying. We have clarified the M&M text (lines 556-7 & 562-3) to reflect this.

Whether or not to rarefy is an ongoing debate in the microbiome field (e.g. see https://academic.oup.com/bioinformatics/article/38/9/2389/6536959, https://journals.plos.org/ploscompbiol/article?id=10.1371/journal.pcbi.1003531 and references therein). The decision depends on assumptions about the whole data generating process, ranging from genomic DNA extraction to the final analysis of the sequencing data. In our case have ultimately decided against rarefying as this process could lead to a loss of sensitivity in the data.

Line 501: Selection of isolates according to phylogenetic distribution - was this based on the *gyrB* genotype? I don't think the *gyrB* sequencing was mentioned in the Methods…

The phylogenetic tree in our study was indeed based on *gyrB*. We apologise for the omission of the *gyrB* sequencing method, which seems to have crept in between manuscript versions. This has now been added to the Materials and Methods section (Line 509 of the revised draft).

Line 479: Details on the high-throughput screening protocol should be provided.

We have revised this section of the Materials and Methods to clarify that our high-throughput screening approach used a microplate replicator to screen hundreds of isolates in parallel on large square plates.
