# Supplementary material for: The genotype of barley cultivars influences multiple aspects of their associated microbiota via differential root exudate secretion
Source: PLoS Biol. 2024 Apr 25;22(4):e3002232. doi: 10.1371/journal.pbio.3002232 (PMC11045101; doi:10.1371/journal.pbio.3002232)
Supplement: Attachment — Submitted filename: Response to reviewers comments on first revision.docx [file pbio.3002232.s028.docx]

**Response to reviewers comments on first revision**

We thank both reviewers for their detailed and insightful reviews of our work, which have significantly improved the final manuscript. We address their remaining points below.

**Reviewer #1:**

The authors tried to address the concerns there were about the previous version and this is highly appreciated. Nevertheless, some issues still remain or not totally addressed. Please see below.

Major issues

1. It is probable that it would benefit the storyline if section "Chevallier and Tipple differ in root exudates composition" is presented first in the story. Then the authors can build their story on the fact that they have different exudation patterns, therefore they could differentially shape the microbiome. Then within the exudates, sugars have a pronounced effect on Pseudomonads, etc.

This is an interesting suggestion, and one we considered while drafting the manuscript. However, we found that this led to some sets of experimental data, such as the phenotypic analysis and the luminescent biomarker assays, sitting rather awkwardly in the narrative. Given that this is a subjective decision that does not impact the science in our study, we decided to keep the manuscript in its current order.

2. Regarding section "Primary carbon metabolism is central to Pseudomonas spp. cultivar adaptation", I really think that testing the effect of exudate mix from each cultivar would be beneficial before testing individual metabolites. That will show indeed whether exudates of each cultivar can have an effect on Pseudomonas. If this is not possible for all isolates (which is understandable), a subset could be tested (with isolates deriving from the 2 cultivars). By subset, some of 42 isolates could be tested - line 233). I cannot see why this is that hard to do. I suspect that a big volume of exudate can be collected by barley and then test effects on Pseudomonas isolates in 96-well plates. That requires some ul per well.

Despite several attempts we were unable to reliably measure microbial growth on either Tipple or Chevallier root exudates in 96 well plates. This was largely due to the difficulty in concentrating exudates enough to enable measurable OD_600_ readings before evaporation compromised the experiment. Consequently, we produced a set of agar plates containing Chevallier and Tipple exudates and observed the growth of our 42 sequenced isolates on these. The results of these assays are now included in as Supplementary Figure 5 and discussed on lines 247-251 of the manuscript.

3. Line 245, in their reply to comment on previous version, the authors mention that SBW25 is isolate from sugar beet, therefore its use here and any effects might be non-specific for the host and not relevant for the cultivars used here. Then why to use SBW25 and its mutants?

SBW25 was chosen for these experiments because it is a well-studied, genetically tractable *Pseudomonas* isolate that robustly colonises the rhizosphere of cereal plants, and contains copies of all of the differentially present genes in our sequenced isolates that were chosen for additional analysis. The fact that SBW25 is not a barley isolate limits the agricultural relevance of any plant phenotypes it may induce, but does not restrict its use as a well-defined experimental model for the comparison of different genes to one another. Using this strain as a baseline for our lab experiments studying the relative importance of different genes for rhizosphere colonisation meant that all eight mutants can be compared to one another in a single experiment.

4. Line 262, I still feel that info on function of the genes would benefit readability and function of figure. Now it's mostly showing how many isolates have a given gene, without knowing its function. Even a colored dot indicative of activity (e.g. transporter, secretion system, etc) on top of each bar could work.

We have modified the figure and updated the legend as suggested.

5.	Line 402, what about the effect on the plant (comment in previous version as well)? Does this difference or lack of in colonization have an influence on the effect of the bacteria on the plant?

We did not examine the impact of the tested genes on seedling growth in our experiments, so we cannot comment on this here. As we state above (point 3), SBW25 is not a barley isolate, which limits its relevance in growth-promotion experiments. However, looking at the effect of key genes on plant growth in a barley PGPR would be an interesting area for future study.

Minor issues

1. The title reads too general. Other than the first figure where the overall microbiome is assessed, in the rest of the manuscript the focus in on Pseudomonas sp. Therefore the authors should mention Pseudomonas and not microbiota in general.

As our manuscript does show major shifts in total microbiome abundance between cultivars, and also how the composition of the whole microbiome impacts on barley cultivar growth, we would prefer to keep the title as it is.

2. Lines 84-85: The background research question is pretty general. How does this advance our knowledge from what Bulgarelli already showed also in Barley (lines 66-67)?

The Bulgarelli study focussed on how genetic loci in barley led to shifts in total microbiome abundance, and did not really address microbiome shifts below the genus/species complex level. Our study focusses more closely on the microbiology of individual soil species, and less on plant genetics. We feel that the two studies nicely complement each other, rather than retreading the same ground.

3. Lines 106-107: how is it possible that a microbiome is still present after sterilisation?

The peat component of Cereal Mix is not sterilised prior to mixing. As we state in the text, ‘*While the soil in this compost is sterilised to kill soil-borne cereal pathogens, Cereal Mix nonetheless retains a complex microbiome derived from the peat component.*’

Line 131: Can 90% abundance of two genera be representative of field conditions, where more diversity is expected?

The text at line 131 and the associated graph only refer to the 30 most abundant genera, hence in the overall population of these genera do not necessarily represent 90% of the total microbial abundance.

Line 161: observed richness? And df isn't it supposed to be number of replicates per group minus 1?

Observed richness, also known as observed species or species richness, is the simplest alpha diversity index. It considers the number of different taxa (in this study ASVs) present in a given sample. Regarding the df (degrees of freedom) value, this displays the degrees of freedom for the independent variable (first number, the number of levels in the variable minus 1), and the degrees of freedom for the residuals (second number, the total number of observations minus one and minus the number of levels in the independent variables). This is now clarified in the text on line 149.

4. Line 168: in both cultivars Pseudomonas is among most abundant genera. Not sure about term "preferentially".

We have changed ‘preferentially’ to ‘differentially’ for clarity.

5. Line 175: please indicate how many are isolated from each cultivar.

This information is included in the materials and methods section (line 493-494 – ‘*20 isolates were randomly selected per plant, for a total of 120 isolates per cultivar across two biologically independent experiments.*’)

6. Line 179: Pseudomonas is genus not species.

This has been changed to ‘microorganisms’ to avoid confusion.

7. Line 197: please add statistics in Suppl. Figure S3.

The statistics for Figure S3 are presented in the Chi Squared analysis that is shown below each graph, where the p-values are represented as a heatmap.

8. Line 255, Illumina can be omitted.

This has been changed as requested.

9. Line 275, what the authors know is hexoses not specifically glucose, right?

Our GC/MS analysis indicates that _D_-glucose is enriched in Tipple root exudates (see Supplementary Table 2 and line 223 of the main text), so we believe the text on line 275 (now line 284) is accurate as it stands.

10. Lines 329-331: But why is it important to understand those ecological effects? What is the potential benefit? up to here it sounds like a mere curiosity.

A line has been added to the text to clarify that this is an issue of sustainability.

11. Line 353, the authors should elaborate on why they focused on Pseudomonas before going into the details.

The reasons for choosing *Pseudomonas* as a model in this study are explained on lines 174-177 of the main text. We therefore feel that repeating these here would be redundant.

**Reviewer #2:**

I have now carefully reviewed the original submitted manuscript and this revised version. The authors have sufficiently addressed most of my concerns, with the following exception:

My original review stated, "To interpret ANOVA results, it is mandatory to specify exactly what explanatory variables, covariates, interactions, etc. were included in the model, as well as what method of calculating sums of squares was used (Type I, Type II, etc.). Different variables each have their own F statistic, df and p-value, which should be reported in the text as support instead of a p-value for the ANOVA in general. In simple cases (one-way ANOVA) they may be the same but as written the reader has no way to know that."

In lines 121-163 of the revised version, the authors provide some additional detail (e.g., the degrees of freedom) which only partially addresses my original comment. These paragraphs describe evidence for differences in microbiome composition and alpha diversity between various groups of samples. I still cannot fully interpret these results because the authors still do not provide the actual models being tested in the ANOVA(s). So let me clarify and repeat my request: the Methods section must include the statistical models in full, such as:

"A two-way ANOVA with Type III sums of squares was used to model the alpha diversity metric as a function of Cultivar, Compartment, and the interaction between Cultivar and Compartment". Or "A one-way ANOVA with Type III sums of squares was used to model alpha diversity, with plant Compartment as the explanatory variable; data from both cultivars were pooled for this analysis". Any given F-test for the Compartment effect (for example) would be interpreted differently depending on which of those two models it came from, because the first approach simultaneously controls for the Cultivar effect and allows the Compartment effect to vary between cultivars; in contrast, the second approach simply reports the Compartment effect averaged across all levels of all other variables involved in the study. So, this information is mandatory and must be provided in the Methods for each ANOVA, Kruskal-Wallis, ANOSIM, and ADONIS test that was performed.

The analysis we used was a one-way ANOVA with type I sums of squares. We have clarified these details in the Materials section as requested.

A few other minor comments:

Line 92: Is it appropriate to say that genotype-driven selection in the rhizosphere is "primarily" driven by root exudates when alternative mechanisms were not really explored?

We have changed ‘primarily driven’ to ‘strongly driven’ here for clarity.

Lines 109-111: These add up to 100%, which indicates that there were not any microbiome members from phyla other than these four. This is unusual for a plant microbiome study. What could explain the total absence of organisms from other phyla commonly seen in the rhizosphere, such as Firmicutes/Bacillota and Verrucomicrobiota?

As mentioned in the response to reviewer 1, this figure and the text linked to it only corresponds to the 30 most abundant bacterial or fungal genera. So the graph shows the relative proportions of those 30 most abundant microbes only, and does not visualise the whole community of the microbiome. This does not mean that there isn’t anything else in the samples, but for simplicity we only represent and comment on the most abundant organisms.

Lines 151-153: What is the Krustal test? Perhaps meant to be Kruskal-Wallis?

This should say Kruskal-Wallis. We have corrected the text and thank the reviewer for pointing it out.

Lines 218-219: The use of "log2 fold change" and "log2 abundance" here is quite confusing. When describing the difference between cultivars, the authors report a "log2 fold change" for each of the two cultivars, which implies that the exudate concentration in each cultivar was compared to some shared control or standard, which is not mentioned. In my original review I had asked a similar question about Fig. 3b, to which you confirmed that the log2 fold change *between cultivars* was calculated directly, rather than using a comparison to a shared control or standard. So I am struggling to understand what these two reported log2 fold changes for the cultivars (16.474 for Chevallier, and -12.613 for Tipple) actually are. Is it possible that these numbers are actually the log2-transformed abundances (not fold changes), like what you reported for D-glucose in line 220? I am further confused because the "derivative of phosphoric acid" was described as absent from Tipple, so how was this 0 value transformed into a log2 fold change? Please clarify what these numbers actually are.

In order to reduce the variability between the replicates and to make appropriate comparisons, the dataset was normalised and the median of all samples was established as a baseline for their abundance (aka peak intensity). The data shown here is the log_2_ normalised abundance in comparison to this median. The data we present shows a comparative increase/decrease in fold change for each compound, i.e. compound x has increased 3 fold in sample type 1 so must have decreased 3 fold in sample type 2. We felt that this approach was more appropriate than the alternative (designating one sample as a ‘control’ and comparing this to the other to find a fold change). The text has been amended to clarify this point.
